# Polymorphisms of methylenetetrahydrofolate reductase (MTHFR) and susceptibility to pediatric acute lymphoblastic leukemia in a German study population

**DOI:** 10.1186/1471-2350-6-23

**Published:** 2005-05-27

**Authors:** Eckart Schnakenberg, Andrea Mehles, Gunnar Cario, Klaus Rehe, Kathrin Seidemann, Brigitte Schlegelberger, Holger A Elsner, Karl H Welte, Martin Schrappe, Martin Stanulla

**Affiliations:** 1 Institute for Pharmacogenetic and Genetic Disposition, Langenhagen, Germany; 2 Children's Hospital, Pediatric Hematology and Oncology, Hannover Medical School, Germany; 3 Institute of Cell and Molecular Pathology, Hannover Medical School, Germany; 4 Department of Transfusion Medicine, Hannover Medical School, Germany

## Abstract

**Background:**

Methylenetetrahydrofolate reductase (MTHFR) has a major impact on the regulation of the folic acid pathway due to conversion of 5,10-methylenetetrahydrofolate (methylene-THF) to 5-methyl-THF. Two common polymorphisms (677C>T and 1298A>C) in the gene coding for MTHFR have been shown to reduce MTHFR enzyme activity and were associated with the susceptibility to different disorders, including vascular disease, neural tube defects and lymphoid malignancies. Studies on the role of these polymorphisms in the susceptibility to acute lymphoblastic leukemia (ALL) led to discrepant results.

**Methods:**

We retrospectively evaluated the association of the *MTHFR *677C>T and 1298A>C polymorphisms with pediatric ALL by genotyping a study sample of 443 ALL patients consecutively enrolled onto the German multicenter trial ALL-BFM 2000 and 379 healthy controls. We calculated odds ratios of *MTHFR *genotypes based on the *MTHFR *677C>T and 1298A>C polymorphisms to examine if one or both of these polymorphisms are associated with pediatric ALL.

**Results:**

No significant associations between specific *MTHFR *variants or combinations of variants and risk of ALL were observed neither in the total patient group nor in analyses stratified by gender, age at diagnosis, DNA index, immunophenotype, or TEL/AML1 rearrangement.

**Conclusion:**

Our findings suggest that the *MTHFR *677C>T and 1298A>C gene variants do not have a major influence on the susceptibility to pediatric ALL in the German population.

## Background

Methylenetetrahydrofolate reductase (MTHFR) has a major impact on the regulation of the folic acid pathway due to the conversion of 5,10-methylenetetrahydrofolate (methylene-THF) to 5-methyl-THF. Two common non-synonymous coding region polymorphisms (677C>T and 1298A>C) in the *MTHFR *gene were shown to confer reduced enzyme activity in in vitro assays leading to a decreased pool of methyl-THF and the *MTHFR *variant 677C>T was associated with an increased risk of hyperhomocysteinemia, particularly in folate-deficient states [1-4]. With regard to the *MTHFR *677C>T polymorphism, results from in vitro assays showed a decrease in enzyme activity to 65% for the heterozygous and to 30% for the homozygous state of the 677T variant [5]. For the 1298A>C polymorphism, enzyme activity in vitro is decreased in homozygous variants and, to a lesser extent, in heterozygotes compared with those homozygous for the wild-type allele [6,7]. It is supposed that variants (677TT/1298AA or 677CC/1298CC) of *MTHFR *increase the level of methylene-THF leading to a subsequent reduction of uracil in DNA. The reduction of uracil decreases the amount of misincorporations of uracil instead of thymidine into DNA. This may protect DNA from double-strand breaks and, consequently, from chromosomal alterations [1,8]. Furthermore,it is assumed that DNA methylation via MTHFR is an important epigenetic feature that modulates DNA methylation status through interaction with folate status [1,9]. Consequently, *MTHFR *variants are discussed to influence disease processes and several studies in the literature reported on reduced MTHFR enzyme activity and the susceptibility to different disorders, including vascular disease, neural tube defects and lymphoid malignancies [1,2,5,7,8,10-13]. Lymphoid malignancies arise as a consequence of point mutations, chromosomal rearrangements (e.g., chromosomal translocations), and epigenetic alterations in hematopoietic cells making the variant *MTHFR *an interesting candidate gene for studies on leukemogenesis [14]. However, especially with regard to susceptibility to acute lymphoblastic leukemia (ALL), previous studies led to discrepant results. Although both variants 677T and 1298C have been reported to decrease susceptibility to ALL in children and adults [8,10,11,13], other investigators did not support such associations [15,16]. Moreover, the protective effect of *MTHFR *variants was present only in children before folic acid supplement in pregnancy has been recommended [17]. These results suggest a role for gene-environment interactions in the association of *MTHFR *with ALL.

In the present study, we analyzed the association of *MTHFR *variants with pediatric ALL in a German study sample including 443 ALL patients and 379 healthy controls.

## Methods

### Patients and controls

The present study used data and specimens derived from patients of the ongoing ALL-BFM 2000 trial that is conducted by the Berlin-Frankfurt-Münster (BFM) study group and enrolls pediatric patients from 1 year up to 18 years of age with a diagnosis of ALL from 84 different treatment centers in Germany, Austria, and Switzerland [18]. From July 1999 until the end of February 2001, 497 patients of up to 18 years of age were enrolled onto the clinical trial after informed consent was obtained from the parents or legal guardians. The diagnosis was established in our central reference laboratory by morphological FAB criteria and cytochemistry when at least 25% lymphoblasts were present in the bone marrow, or when blasts were present in the peripheral blood. The assessments of immunophenotype, cellular DNA content, and positivity for TEL/AML1, BCR/ABL, and MLL/AF4 fusion genes were done as described previously [19,20]. Patients were included into the present study, when biological material was available at the study center leading to a study population of 443 patients (89.7% of the entire study population) who with regard to clinical characteristics did not significantly differ from the entire study population of 497 patients. Controls (n = 379) consisted of blood samples from healthy blood donors (aged between 18–68 years), with no history of malignant neoplastic disease. They were derived through the Department of Transfusion Medicine, Hannover Medical School, Hannover, Germany, and the Clinic Center of Bremen, Bremen, Germany. Individuals included in the present study were of Caucasian descent. The study was approved by the local ethics committee.

### Genotyping

DNA was extracted from peripheral blood or bone marrow samples by using the QIAamp DNA Blood Midi Kit (Qiagen GmbH, Hilden, Germany). Depending on availability, either tumor material or remission samples were used. Analyses of *MTHFR *variants were performed on a LightCycler^® ^instrument (Roche Diagnostics, Mannheim Germany) using a commercial real-time assay (Artus Biotech, Hamburg, Germany) according to the manufacturer's instructions. Both variants *MTHFR *677C>T and 1298A>C were analyzed by simultaneous amplification in a single real-time PCR run. Melting curve analyses were performed for both variants after PCR amplification. No differences in the distribution of *MTHFR *variants were observed between tumor and remission samples (data not shown).

### Statistical analysis

The association of *MTHFR *variants with risk of disease was examined by use of unconditional logistic regression analysis to calculate odds ratios (OR) and their 95% confidence intervals (CI). *P *values of <0.05 were considered statistically significant. The *MTHFR *variant was used as a categorical variable in these analyses. The expected frequency of *MTHFR *variants in controls was analyzed by the Hardy-Weinberg equilibrium test. The SPSS statistical package (SPSS Inc., Chicago, IL) was used for computerized calculations.

## Results and Discussion

Table 1 shows the distribution of clinical characteristics in the study sample of 443 childhood ALL patients from ALL-BFM 2000. Distribution of *MTHFR *variants within patients and controls are summarized in Table 2. In our control population of 379 healthy individuals the frequencies of *MTHFR *variants 677C>T and 1298A>C were in Hardy-Weinberg equilibrium (677: χ^2 ^= 1.81, 1298: χ^2 ^= 0.05; *P *> 0.05) and demonstrated strong linkage disequilibrium (P < 0.001). None of the haplotypes was observed more frequently in the ALL group (Tab. 2). *MTHFR *variants that were previously reported to confer a reduced risk of ALL were similarly distributed in the patient and control group. The frequency of the variant *MTHFR *677T was 0.314 in controls and 0.333 in children with ALL. Ogino et al. reported a frequency of 0.320 in a meta-analysis including 5389 individuals [21]. There was also no significant different distribution of the *MTHFR *1298C variant between our patient and control groups. The 1298C variant occurs with a frequency of 0.367 in controls and 0.314 in patients with ALL. Ogino et al. reported 0.308 in their meta-analysis [21]. The calculated OR and their respective 95% CI are also shown in Table 2. No significant associations between specific *MTHFR *variants or combinations of *MTHFR *variants and risk of ALL were observed neither in the total patient group nor in analyses stratified by gender, age at diagnosis, DNA index, immunophenotype, or TEL/AML1 rearrangement (stratified analyses not shown; strata as shown in Table 1).

With regard to ALL, our results are in contrast to previous findings and do not support the assumption that higher methylene-THF levels due to *MTHFR *variants lead to a decreased risk of ALL in our study population [8,10,11,13]. There are different explanations for these diverging results. The present study included pediatric patients from Germany. The above mentioned studies included adult patients from United Kingdom [8] and Italy [13] but also pediatric patients from United Kingdom [10] or Brazil [11]. These latter two studies on the *MTHFR *677C>T and 1298A>C polymorphisms in childhood ALL included patients diagnosed between 1992–1998 and 1991–2000, respectively, while our study included pediatric ALL patients diagnosed after July 1999. Thus, probably a large proportion of patients analyzed in the studies by Wiemels et al. [10] and Franco et al. [11] were born before folic acid supplementation in pregnancy was recommended. In a Canadian study, Krajinovic et al. reported that the protective effect of *MTHFR *variants was accentuated and present only in children born before 1996 [17]. This may explain the diverging results with regard to pediatric ALL and is further supported by a recent study of Balta and colleagues from Turkey, where in 142 pediatric ALL patients diagnosed between February 2000 and February 2002, no significant association between the *MTHFR *677C>T polymorphism and ALL was detected [16]. However, the impact of folate metabolism on the risk of ALL may also vary from population to population because of additional gene-environment and gene-gene interactions. For example, in our study population we cannot control for differences in folate status as we have not collected information on dietary intake. Thus, a potentially existing association of *MTHFR *variants and ALL may be masked by the inability to control for the individual folate status in our study population. Furthermore, we cannot exclude potential gene-gene interactions. As several polymorphic key enzymes are involved in folate metabolism, a more comprehensive approach would clearly yield more precise estimates of the association of folate metabolism with risk of ALL. As an additional explanation for diverging results, specifically in subgroups with chromosomal translocations, the potential importance of parental variants and folate status with respect to an in utero pathogenesis of ALL (e.g., MLL/AF4 and TEL/AML1 fusion gene-positive ALL) should be considered [22]. Lastly, our results may simply be due to chance and small patient numbers. However, with regard to sample size, our study exceeds most other studies reported in the literature, so far. In conclusion, our findings suggest that the *MTFHR *gene variants analyzed here do not have a major influence on the susceptibility to pediatric ALL in the German population. However, potential effects in rare specific ALL subgroups cannot be excluded.

## Conclusion

We retrospectively evaluated the association of the *MTHFR *677C>T and 1298A>C polymorphisms with childhood ALL by analyzing a study sample of 443 ALL patients consecutively enrolled onto the German multicenter trial ALL-BFM 2000 and 379 healthy controls. No significant associations between specific *MTHFR *variants or combinations of *MTHFR *variants and risk of ALL were observed neither in the total patient group nor in analyses stratified by gender, age at diagnosis, DNA index, immunophenotype, or TEL/AML1 rearrangement. Our findings suggest that the *MTHFR *677C>T and 1298A>C gene variants do not have a major influence on the susceptibility to pediatric ALL in the German population.

## Competing interests

The author(s) declare that they have no competing interests.

## Authors' contributions

All authors were responsible for the concept of the study. ES and MSt coordinated the study. All authors were involved in sample collection, DNA preparation, genotyping and interpretation of the analyses. MSt and ES did the statistical analyses, drew-up the tables and prepared the manuscript with advice from the other authors. MSch is the chairman of the ALL-BFM 2000 study.

## Pre-publication history

The pre-publication history for this paper can be accessed here:



## Figures and Tables

**Table 1:** Clinical characteristics of patients from trial ALL-BFM 2000

	Patients ALL-BFM 2000 (n = 443)
	Number of subjects (%)
Gender	
Male	268 (60.5)
Female	175 (39.5)
	
Age at diagnosis (years)	
< 1	-
1-< 6	264 (59.6)
6-< 10	80 (18.1)
≥ 10	99 (22.3)
	
DNA index^a^	
< 1.16	258 (58.2)
≥ 1.16	48 (10.8)
no result	137 (30.9)
TEL/AML1 pos.	90 (20.3)
neg.	313 (70.7)
unknown	40 (9.0)
	
BCR/ABL pos.	9 (2.0)
neg.	412 (93.0)
unknown	22 (5.0)
	
MLL/AF4 pos.	1 (0.2)
neg.	394 (88.9)
unknown	48 (9.9)
	
Immunophenotype	
Precursor B	344 (77.7)
T	81 (18.3)
Bipenotypic	4 (0.9)
Unknown	14 (3.2)

^a^Ratio of DNA content of leukemic G^0^/G^1 ^cells to normal diploid lymphocytes.

**Table 2:** Distribution of MTHFR variants in cases and controls and their association with childhood acute lymphoblastic leukemia in patients from trial ALL-BFM 2000

*MTHFR*	Cases (n = 443) Number (%)		Controls (n = 379) Number (%)		Odds ratio (95% CI)	*P*
677 CC/1298 AA	49	(11.1)	45	(11.9)	1.00^a^	
677 CT/1298 AA	101	(22.8)	67	(17.7)	1.38 (0.83–2.30)	0.21
677 TT/1298 AA	44	(9.9)	41	(10.8)	0.99 (0.55–1.77)	0.96
677 CC/1298 AC	101	(22.8)	87	(23.0)	1.07 (0.65–1.75)	0.80
677 CT/1298 AC	100	(22.6)	85	(22.4)	1.08 (0.66–1.78)	0.76
677 TT/1298 AC	3	(0.7)	2	(0.5)	1.38 (0.22–8.63)	0.73
677 CC/1298 CC	45	(10.2)	52	(13.7)	0.79 (0.45–1.40)	0.43
677 CT/1298 CC	n.d.		n.d.		-	-
677 TT/1298 CC	n.d.		n.d.		-	-

n.d., not detected; CI, confidence interval; ^a^reference category
